# A Rare Case of Tricuspid Atresia Absent Pulmonary Valve Diagnosed on Fetal Echocardiography

**DOI:** 10.1016/j.case.2023.09.002

**Published:** 2023-10-30

**Authors:** Wesam Sourour, Shannon K. Powell

**Affiliations:** Department of Pediatric Cardiology, Children's Hospital New Orleans, Louisiana State University Health Sciences Center, New Orleans, Louisiana

**Keywords:** Tricuspid atresia absent pulmonary valve, Fetal echocardiography, Prenatal diagnosis

## Abstract

• TA absent PV is a rare congenital heart anomaly.
• TA absent PV is associated with a poor prognosis.
• TA absent PV can be successfully diagnosed on fetal echocardiography.

## Graphical abstract

**Figure:**
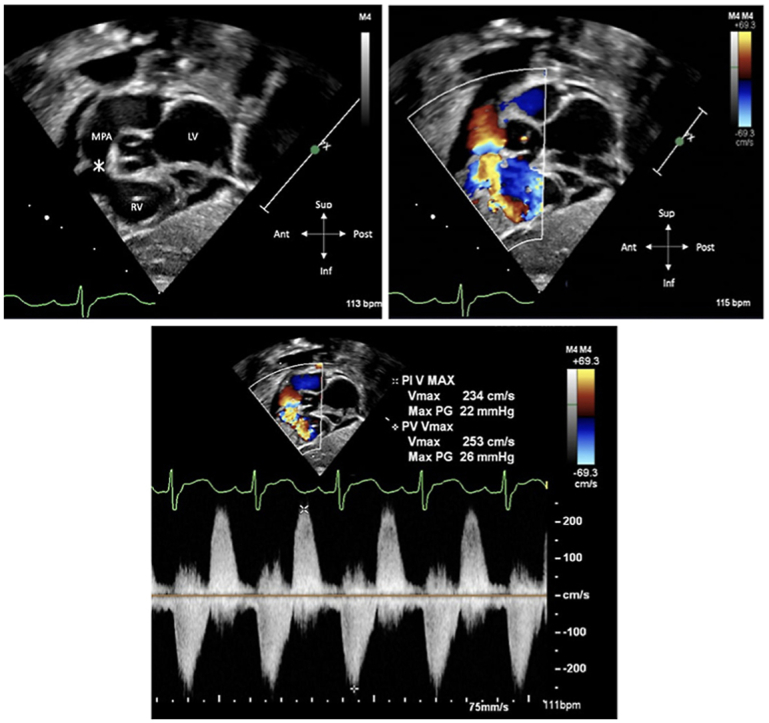

## Introduction

Tricuspid atresia (TA) absent pulmonary valve (PV) with an intact ventricular septum is a very unusual anomaly that is associated with a poor prognosis. We present a patient with a rare diagnosis of TA absent PV and intact ventricular septum diagnosed on fetal echocardiography.

## Case Presentation

**Figure 1:**
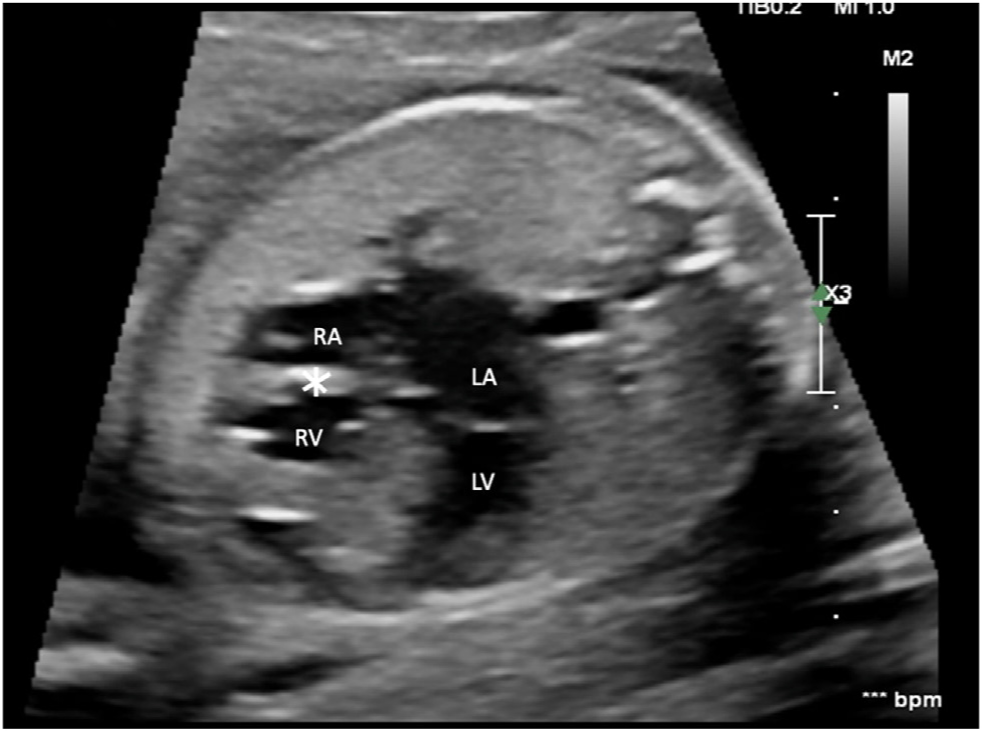
Transabdominal two-dimensional fetal echocardiogram, 4-chamber view, demonstrates a severely hypoplastic, hypertrophied, and aneurysmal RV with an atretic tricuspid valve (*asterisk*). *LA*, Left atrium; *RA*, right atrium.

**Figure 2:**
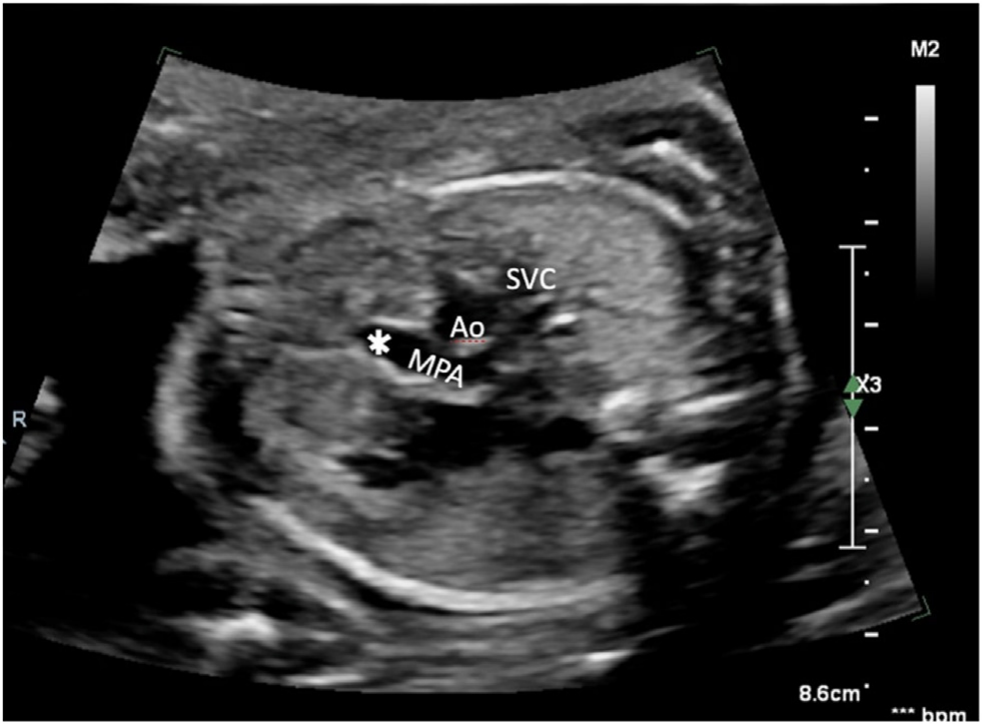
Transabdominal two-dimensional fetal echocardiogram, outflow tract view, demonstrates a normal-sized MPA and no evidence of PV leaflets (*asterisk*). *Ao*, Aorta; *SVC*, superior vena cava.

Our patient was born to a 29-year-old G3 P1 woman who was referred from an outside institution for a second opinion. The anatomy scan was concerning for a “mass in the right ventricle,” and thus they were referred for a fetal echocardiogram and further management. No extracardiac anomalies were noted. The fetal cardiac assessment was completed at 24 weeks of gestation and showed TA with a severely hypoplastic, hypertrophied, and aneurysmal right ventricle (RV) that had diminished function. The ventricular septum was found to be bulging into the left ventricle (LV) (Figure 1, Videos 1 and 2) without obstruction by pulsed-wave Doppler. The great vessels were normally related, but the PV was unable to be identified and to-and-fro flow was noted in the pulmonary outflow tract (Figure 2, Video 3) The main pulmonary artery (MPA) and the right pulmonary artery measured normal in size (the left pulmonary artery was not visualized). Pulsed-wave Doppler interrogation of the umbilical artery demonstrated absent diastolic flow. The ductus venosus flow was also decreased, with atrial contraction. The presumptive diagnosis of TA with normally related great vessels and absent PV syndrome was made.

The rest of the pregnancy was unremarkable, with appropriate interval fetal growth on serial ultrasounds and no evidence of hydrops or arrhythmias.

**Figure 3:**
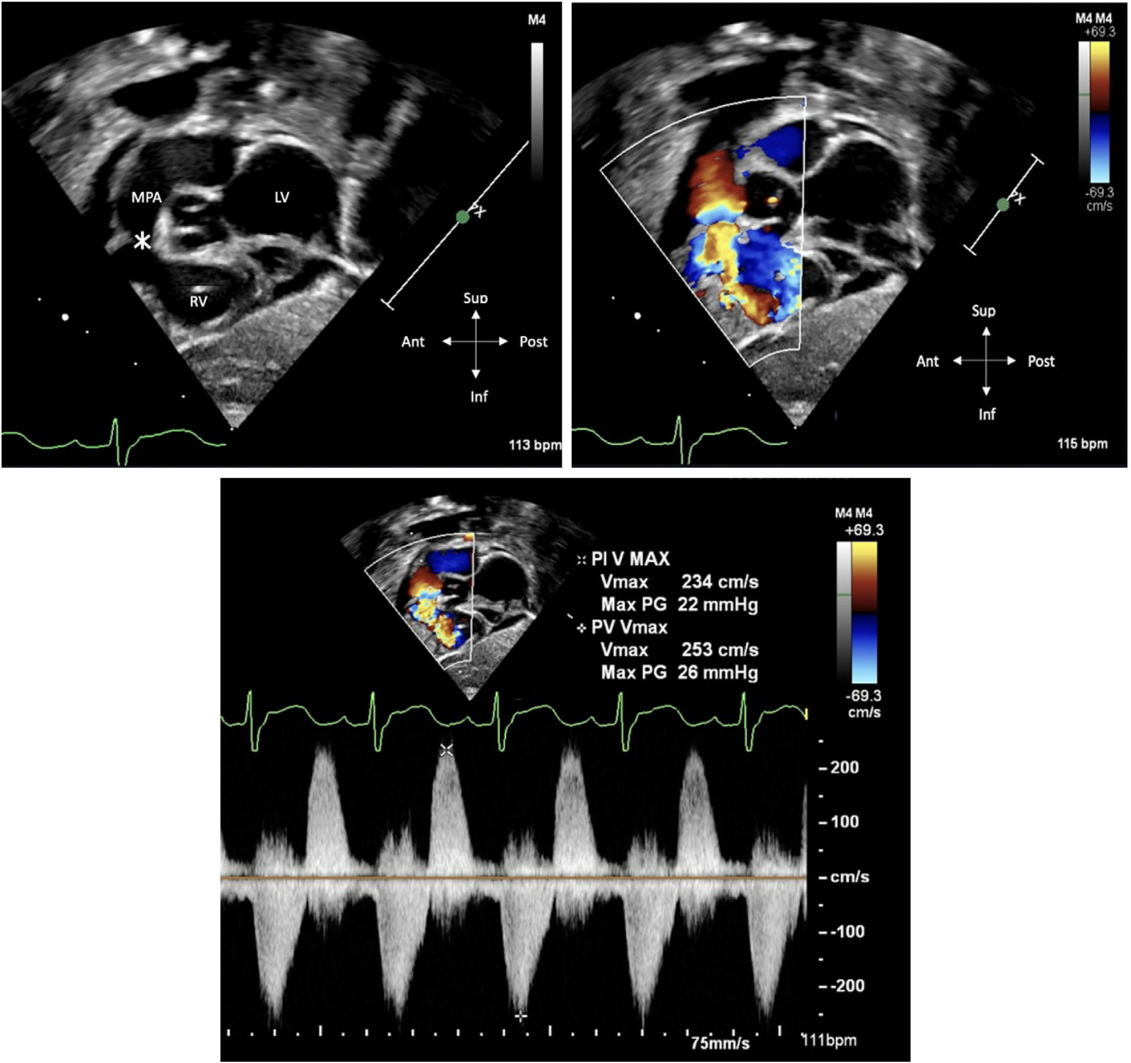
Postnatal two-dimensional transthoracic echocardiogram, subcostal sagittal view, without (*top left*) and with (*top right*) color-flow Doppler, diastolic phase, demonstrates a hypoplastic and hypertrophied RV and no evidence of PV leaflets (*asterisk*) with flow reversal in the MPA due to absence of PV leaflets. Continuous-wave Doppler demonstrates systolic antegrade and diastolic retrograde flow due to absence of valve apparatus (*bottom*).

**Figure 4:**
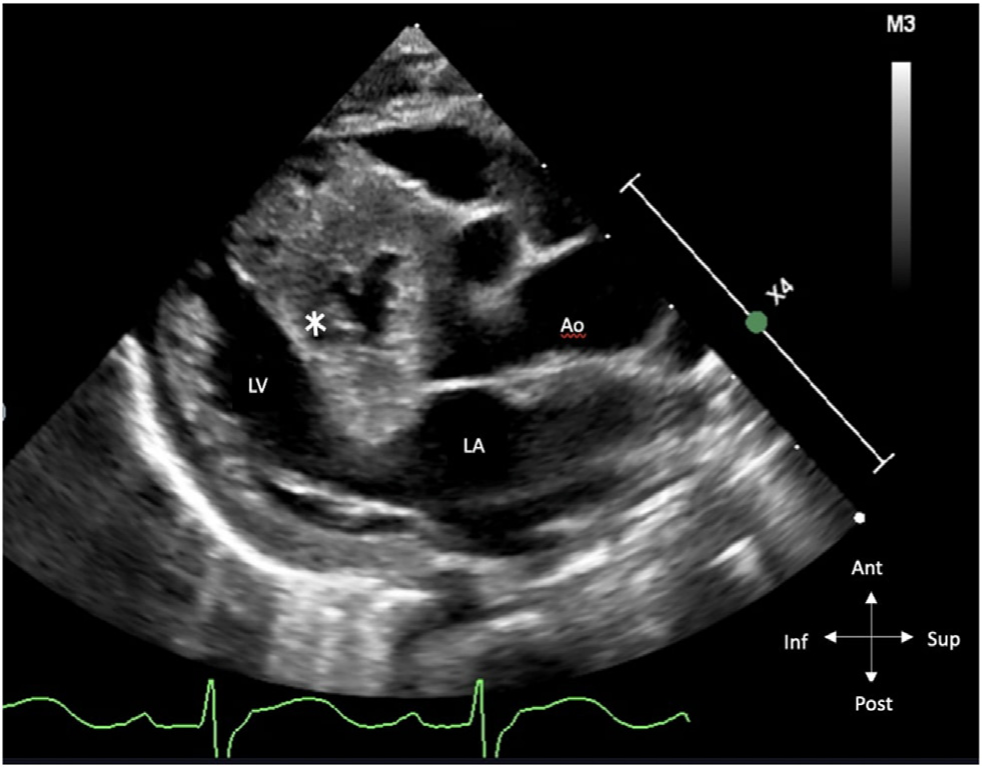
Postnatal two-dimensional transthoracic echocardiogram, parasternal long-axis view, systolic phase, demonstrates a severely hypertrophied and aneurysmal RV with the ventricular septum encroaching into the left ventricular outflow tract (*asterisk*). *Ao*, Aorta.

**Figure 5:**
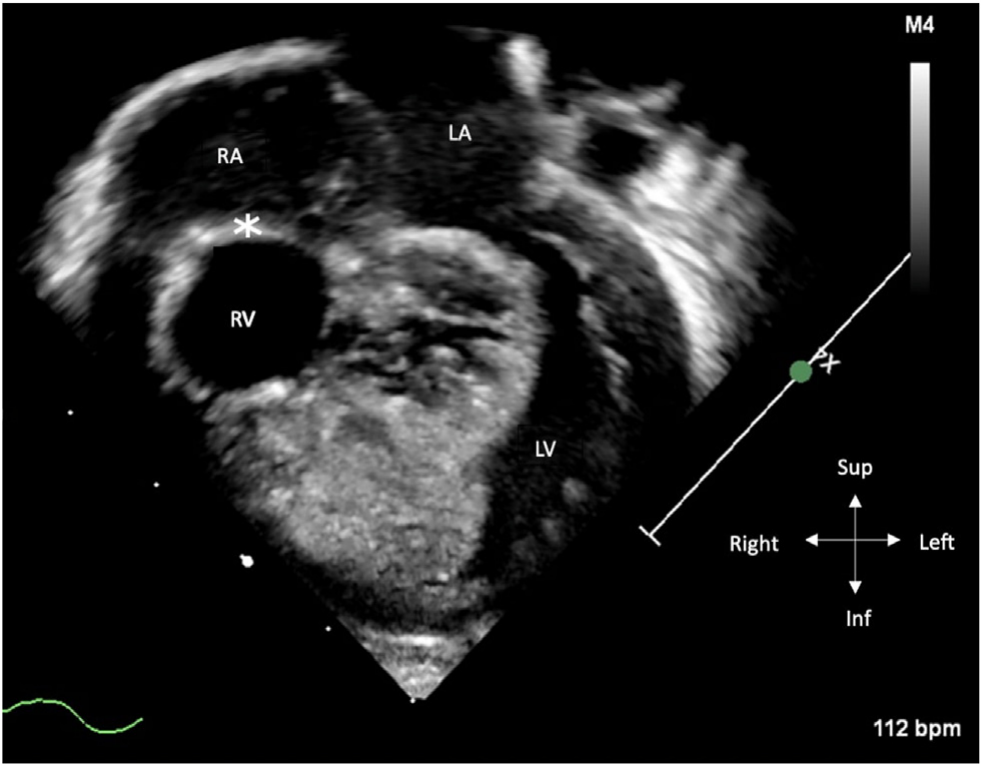
Postnatal two-dimensional transthoracic echocardiogram, apical 4-chamber view, diastolic phase, demonstrates a severely hypertrophied and aneurysmal RV, the ventricular septum encroaching into the LV, and the membranous TA (*asterisk*).

The mother underwent scheduled induction of labor at 39 weeks. The patient was born weighing 3.32 kg and was admitted to the cardiac intensive care unit on a prostaglandin infusion to maintain ductal patency. A postnatal transthoracic echocardiogram confirmed the fetal echocardiogram findings. It demonstrated membranous TA with absent PV, intact ventricular septum, and an aneurysmal RV in addition to a large tortuous patent ductus arteriosus (PDA). The RV septal aneurysm protruded markedly into the left ventricular outflow tract but still did not produce a significant gradient. (Figure 3, Figure 4, Figure 5, Video 4, Video 5, Video 6, Video 7).

**Figure 6:**
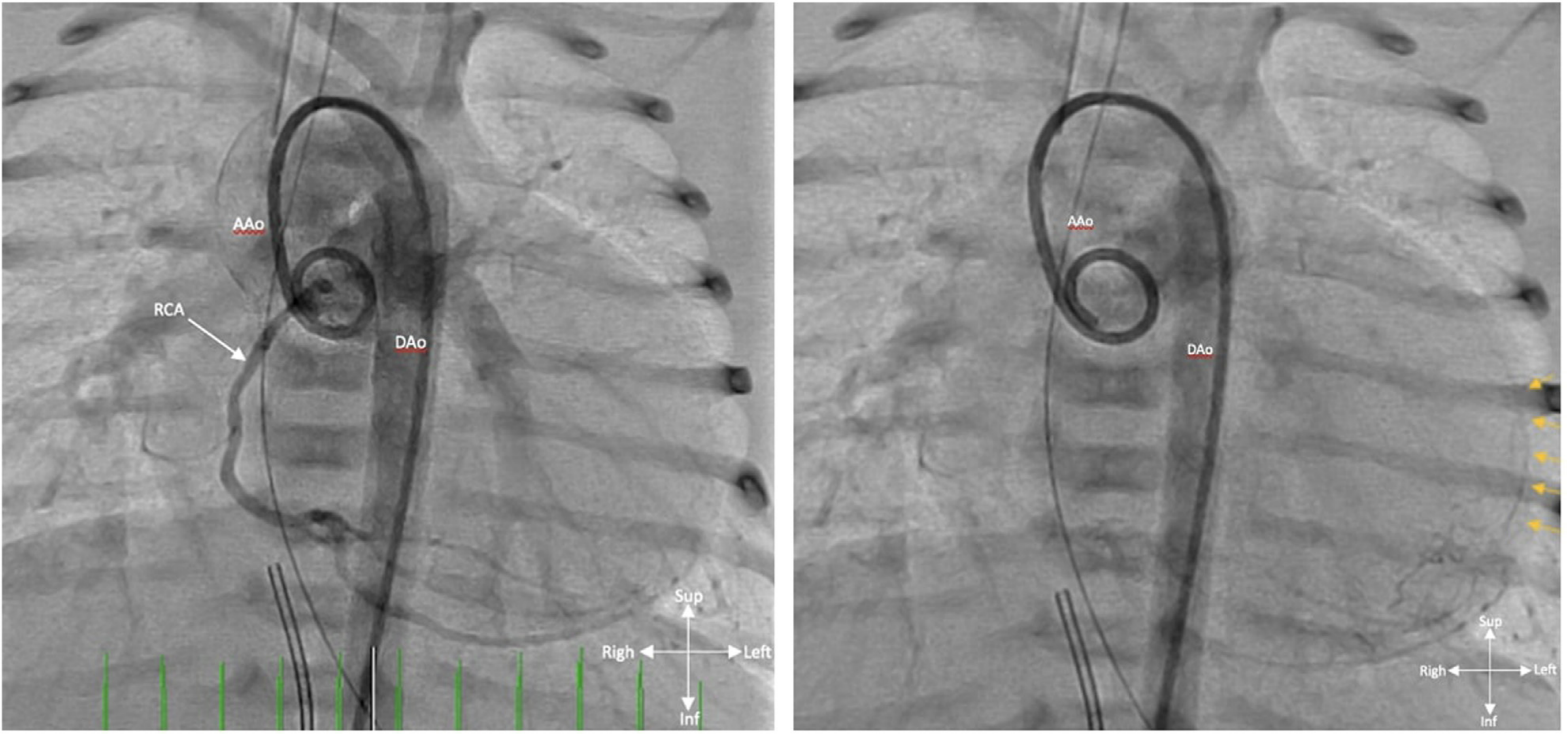
Invasive contrast aortogram performed with a 4 French pigtail catheter positioned in the aortic root, straight anterior-posterior view, demonstrates the normal right coronary artery (*white arrow*) with a right dominant pattern and clearly visualized PDA. No definitive LCA is seen (*leftpanel*). Delayed imaging (*rightpanel*) demonstrates faint retrograde filling in the LAD artery (*orange arrows*). *AAo*, Ascending aorta; *DAo*, descending aorta.

Due to the known association with coronary artery abnormalities in this rare condition, the patient underwent a diagnostic cardiac catheterization with angiography, which demonstrated a right coronary dominant circulation without a definitive left coronary artery (LCA), which was concerning for RV-dependent left coronary sinusoids. (Figure 6, Video 8). This was also supported by faint filling of the left anterior descending artery (LAD) seen from the right coronary artery injection (Figure 6). Unfortunately, due to the tortuosity of the PDA, the RV was unable to be accessed during the procedure for an RV contrast injection and the PDA was unable to be stented. The ratio of total pulmonary blood flow to the total systemic blood flow (Qp:Qs) was found to be 4:1. Preoperatively, the neonate developed pneumatosis and concerns for necrotizing enterocolitis that required medical therapy.

Since the PDA was unable to be stented, the decision was made to proceed with initial surgical intervention via MPA ligation and placement of a central shunt. A ventricular septal defect was also created to support the RV-dependent left coronary system due to the anatomical lack of inflow to the RV and the need to ligate the MPA with the central shunt. There was consideration given to sending the patient for heart transplant postoperatively. Intraoperatively, it was noted that the LCA was small, with an intramural segment that was unroofed. The LAD was unable to be probed surgically and therefore thought to be supplied by the RV through communications that were not visualized.

The postoperative course was complicated by second-degree atrioventricular block with 3:1 conduction and a low ventricular escape rate that required pacing initially before conduction returned to sinus rhythm in subsequent days. However, the rhythm eventually progressed back to second- and even third-degree heart block and led to a cardiac arrest requiring cannulation on to extracorporeal membrane oxygenation (ECMO). Postarrest electroencephalogram demonstrated multifocal seizure activity. The patient was able to be weaned off ECMO 4 days later but went on to develop renal failure with anuria and fluid overload after delayed sternal closure, suspected to be from restrictive cardiac physiology. The chest was reopened and peritoneal dialysis was started. Several days later, the patient sustained a second cardiac arrest from a tachyarrhythmia requiring ECMO with resultant severely diminished cardiac function by echocardiogram and global hypoxic-ischemic encephalopathy by head ultrasound. Ultimately, compassionate extubation was performed secondary to widespread ischemic organ damage at 7 weeks of age.

## Discussion

Tricuspid atresia with an intact ventricular septum is a rare form of congenital heart disease. This was initially described by Marin-Garcia *et al.*^1^ in 1973; however, more cases are continuing to be described in the literature. A recent study of 62 cases reported in the literature found that 89% of prenatally diagnosed cases are live births, typically without associated chromosomal anomalies.^2^

The constellation of findings for this disease are summarized by Litovsky *et al.*^3^ This includes absent tricuspid valve with stenotic or dysplastic leaflets, redundant aneurysmal septum primum, and patent foramen ovale or ostium secundum atrial septal defect, ventricular septal abnormalities including disarray of myofibers or sinusoids, abnormal RV free-wall myocardium, absence of PV leaflets, and primarily right-sided coronary artery abnormalities. It should be noted, however, that a subset of infants may present with tricuspid stenosis rather than atresia, suggesting that this lesion may present as a spectrum with varying degrees of severity.

The etiology is unknown; however, the primary cause is thought to be secondary to failed development of the PV. This leads to antegrade flow in systole and retrograde flow in diastole, which ultimately results in delayed opening and early closure of the tricuspid valve. This flow may elevate the RV end-diastolic pressure and interfere with the normal function of the tricuspid valve that leads to the tricuspid stenosis or atresia.^2^^,^^4^

Tricuspid atresia with absent PV is not associated with pulmonary artery dilation, as demonstrated in our patient. Volume overload due to patency of the ductus is limited to the RV and allows the stroke volume to run off from the RV into the systemic circulation, sparing the pulmonary arteries from volume overload and subsequent dilation.^5^, ^6^, ^7^

Management options include referral for heart transplantation or surgical palliation with creation of a systemic to pulmonary shunt; however, prognosis, while continuing to show improvement, remains poor, with a mortality rate ranging from 33% to 44%.^1^^,^^2^ Patients with coronary artery anomalies have a higher mortality rate.^2^ Thus, in addition to the already poor prognosis of this lesion, our patient’s abnormal coronary anatomy was an additional risk factor for a poor outcome. Furthermore, our case was unique compared to those reported in the literature as the coronary artery anomaly seen was a notably small LCA and an LAD fed by RV sinusoids rather than the more commonly reported right coronary abnormalities.

The management of the MPA for patients undergoing single-ventricle palliation remains controversial. A recent review of the literature by Kawasaki *et al.*^2^ of 57 live cases reported in the literature with TA absent PV found that only 14 of them completed Fontan palliation. Of note, multiple patients received heart transplantation and were alive with a shunt or were awaiting their Fontan palliation at the time of publication. The management of the MPA was only described in 7 cases in the study. The MPA was found to be ligated in 4 patients, partially closed in 2 patients, and not ligated in 1 patient.^2^ Unfortunately, there are insufficient studies to evaluate the hemodynamic effects of MPA ligation in this patient population.

## Conclusion

Tricuspid atresia absent PV with an intact ventricular septum is a rare congenital heart lesion. The pathology of this lesion results in a unique echocardiographic appearance. Providers and sonographers must be cognizant of this heart lesion to allow for improved fetal diagnostic accuracy and counseling as well as appropriate postnatal management given the relatively high mortality rates.

## Ethics Statement

The authors declare that the work described has been carried out in accordance with The Code of Ethics of the World Medical Association (Declaration of Helsinki) for experiments involving humans.

## Consent Statement

The authors declare that since this was a noninterventional, retrospective, observational study utilizing deidentified data, informed consent was not required from the patient under an IRB exemption status.

## Funding Statement

The authors declare that this report did not receive any specific grant from funding agencies in the public, commercial, or not-for-profit sectors.

## Disclosure Statement

The authors report no conflict of interest.

## Supplementary Data

Video 1 Transabdominal two-dimensional fetal echocardiogram, outflow tract view, demonstrates a normal sized MPA and no evidence of PV leaflets.

Video 2 Transabdominal two-dimensional fetal echocardiogram, 4-chamber view, demonstrates a severely hypoplastic, hypertrophied, and aneurysmal RV with an atretic tricuspid valve.

Video 3 Transabdominal two-dimensional fetal echocardiogram with color-flow Doppler, ductal arch view, demonstrates no evidence of PV leaflets with antegrade systolic flow and retrograde diastolic flow.

Video 4 Postnatal two-dimensional transthoracic echocardiogram, subcostal sagittal view, demonstrates a hypoplastic and hypertrophied RV with no evidence of PV leaflets and a normal-sized MPA.

Video 5 Postnatal two-dimensional transthoracic echocardiogram, subcostal sagittal view, color-flow Doppler, demonstrates antegrade systolic flow and retrograde diastolic flow.

Video 6 Postnatal two-dimensional transthoracic echocardiogram, apical 4-chamber view, demonstrates a severely hypertrophied and aneurysmal RV, the ventricular septum encroaching into the LV, and the membranous TA.

Video 7 Postnatal two-dimensional TTE, parasternal long-axis view, demonstrates a severely hypertrophied and aneurysmal RV with the ventricular septum encroaching into the LV outflow tract.

Video 8 Invasive contrast aortogram performed with a 4 French pigtail catheter positioned in the aortic root, straight anterior-posterior view, demonstrates a normal appearing ascending, transverse, and descending thoracic aorta with no areas of dilation or coarctation. There is a normal left-sided aortic arch with abnormal branching of the head and neck vessels. The first branch bifurcates into the right and left carotid arteries, the left subclavian artery, and an anomalous right subclavian artery. There is no aortic valve regurgitation. A normal right coronary artery with a right dominant pattern and clearly visualized posterior descending artery is seen. There is evidence of faint retrograde filling in the LAD.
